# Effects of whole body exposure to extremely low frequency electromagnetic fields (ELF-EMF) on serum and liver lipid levels, in the rat

**DOI:** 10.1186/1476-511X-6-31

**Published:** 2007-11-16

**Authors:** Patricia V Torres-Duran, Aldo Ferreira-Hermosillo, Marco A Juarez-Oropeza, David Elias-Viñas, Leticia Verdugo-Diaz

**Affiliations:** 1 Department of Biochemistry, School of Medicine, UNAM. P.O. Box 70159, Mexico, D.F. 04510, Mexico; 2 Department of Biochemistry, School of Medicine, UNAM. Scholarship holder PAPIIT IN218107 and AFINES, Mexico; 3 Department of Electrical Engineering, Section of Bioelectronics, CINVESTAV, IPN, Mexico, D. F., 07360, Mexico; 4 Department of Physiology, School of Medicine, UNAM. P.O. Box 70250, Mexico, D. F. 04510, Mexico

## Abstract

**Backgound:**

The effects of extremely low-frequency electromagnetic fields (ELF-EMF) on the blood serum and liver lipid concentrations of male Wistar rats were assessed.

**Methods:**

Animals were exposed to a single stimulation (2 h) of ELF-EMF (60 Hz, 2.4 mT) or sham-stimulated and thereafter sacrificed at different times (24, 48 or 96 h after beginning the exposure).

**Results:**

Blood lipids showed, at 48 h stimulated animals, a significant increase of cholesterol associated to high density lipoproteins (HDL-C) than those observed at any other studied time. Free fatty acid serum presented at 24 h significant increases in comparison with control group. The other serum lipids, triacylglycerols and total cholesterol did not show differences between groups, at any time evaluated. No statistical differences were shown on total lipids of the liver but total cholesterol was elevated at 24 h with a significant decrease at 96 h (p = 0.026). The ELF-EMF stimulation increased the liver content of lipoperoxides at 24 h.

**Conclusion:**

Single exposures to ELF-EMF increases the serum values of HDL-C, the liver content of lipoperoxides and decreases total cholesterol of the liver. The mechanisms for the effects of ELF-EMF on lipid metabolism are not well understand yet, but could be associated to the nitric oxide synthase EMF-stimulation.

## Background

Some recent epidemiologic studies have suggested that the exposure to extremely low frequency (ELF) electromagnetic fields (EMF) affect human health, because of the incidence of certain types of cancer, depression, and miscarriage have been increased among individuals living or working in environments exposed to such fields [1-3]. Some of these studies have shown associations between exposure to power-frequency (50–60 Hz) magnetic fields and increased health risk [4,5], but other studies have not shown such a link [6]. The results described above are not completely conclusive, since in several cases they are contradictory.

Extremely low-frequency electromagnetic fields exposure is generally believed to be innocuous for human health due to their low-level energy exposition, which is of a magnitude well below that required to affect the metabolic rate of the human body [3,7,8]. However, an increasing number of studies have reported that ELF-EMF exposure is capable to eliciting *in vivo *and *in vitro *bioeffects [9-12]. ELF-EMF exposure has shown to increase oxidative-stress in some models like chick embryos [13], mammalian cultured cells [14], and human erythrocytes [15]. The increased oxidative-stress involves oxidative DNA damage, lipid peroxidation [16], and may cause a number of systemic disturbances [17].

Spreading evidence suggests that environmental and artificial magnetic fields have significant impact on cardiovascular systems of animals and humans [18-21]. Recent studies showed that static magnetic fields decreased arterial baroreflex sensitivity in rabbits [22]. Under pharmacologically induced hypertensive conditions, the exposure to nonuniform MF to rabbits, significantly attenuated the vasoconstriction and suppressed the elevation of blood pressure [23]. In contrast, microwaves increase skin temperature and therefore cause vasodilatation in normal subjects exposed during 30 minutes [24]. Also, human blood platelets exposed *in vitro *to microwaves produced by mobile phones (operating at 900 MHz), increased the thiobarbituric acid reactive substances (TBARS) production and significantly depleted the superoxide dismutase-1 activity [17]. Recently, it has been studied the possibility that heat generation and the activation of the inducible form of nitric oxide (NO) synthase may be the possible causes of the biological effects of EMF exposure [25-27].

On the other hand, beneficial effects of ELF-EMF have also been reported. In diet-induced hypercholesterolemic rabbits, pulsed of EMF lowers total cholesterol and triacylglycerols levels [28]; similar results have been found in rats [29] and mice [30], both fed on control diets. Despite the above mentioned studies, there are not enough data to know the time-course effects of ELF-EMF on lipid and lipoperoxide levels in biological models. Then, the aim of the present study was to investigate, in the rat, the time-course effects of a single ELF-EMF stimulation on serum and liver lipid concentrations, as well as liver lipoperoxide production.

## Results

### Serum lipid concentrations

**Figure 1:**
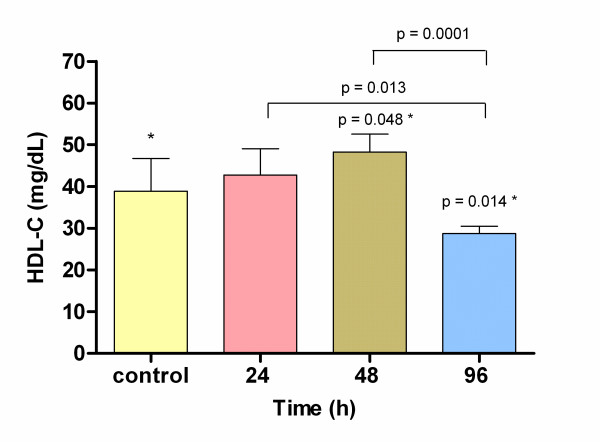
**High density lipoprotein cholesterol serum levels in control group and 24, 48 and 96 h after ELF-EMF exposure**. * Values significantly different in comparison with control group by ANOVA. A significant decrease was observed at 96 h in comparison with 24 and 48 h by ANOVA.

**Figure 2:**
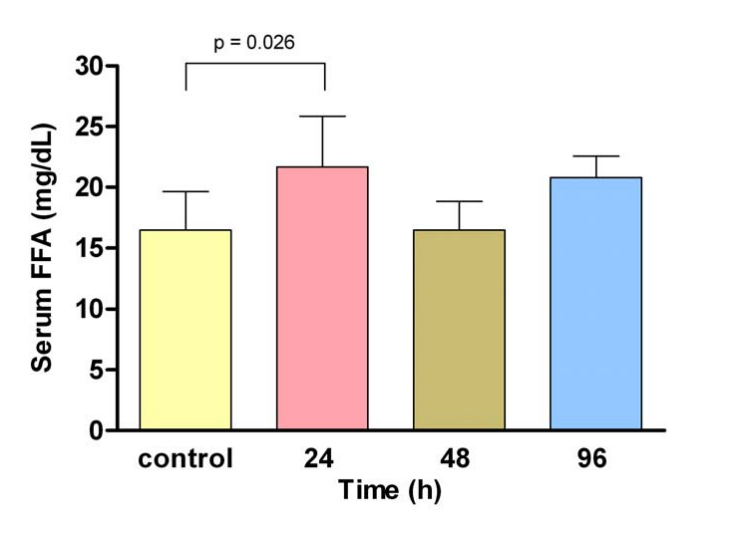
**Free fatty acid serum levels in control group and 24, 48 and 96 h after ELF-EMF exposure**. A significant increase was observed at 24 h in comparison with control group by ANOVA.

There were no significant differences between experimental and control groups for triacylglycerols and total cholesterol serum levels, at any time analyzed. However, 48 h after beginning the treatment, HDL-C concentrations were higher in stimulated rats (48.2 ± 4.3 mg/dL) than those observed in control group (38.7 ± 7.1 mg/dL); however, a significant decrease on HDL-C levels was observed at 96 h in ELF-EMF stimulated rats (24 h 46.1 ± 8.1 mg/dL and 96 h 29.24 ± 2.20 mg/dL) (Fig. 1). On the other hand, FFA serum levels increased at 24 h (20 ± 2.25 mg/dL) with a statistically significant difference respect control (16.6 ± 3 mg/dL; p = 0.026) but no differences were found at 48 or 96 h (Fig. 2).

### Liver analyses

**Figure 3:**
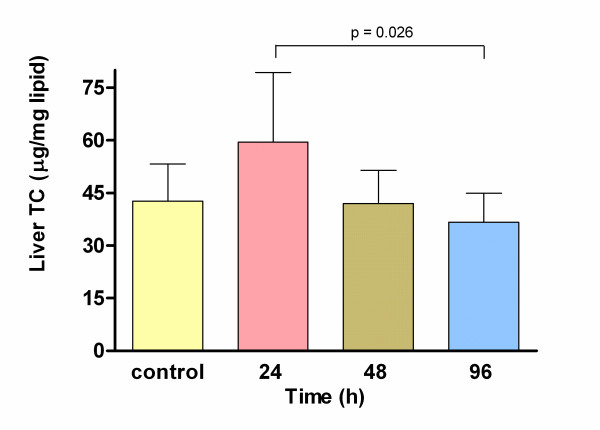
**Total cholesterol liver levels in control group and at 24, 48 and 96 h after ELF-EMF exposure**. A significant decrease was observed at 96 h in comparison with 24 h by ANOVA.

**Figure 4:**
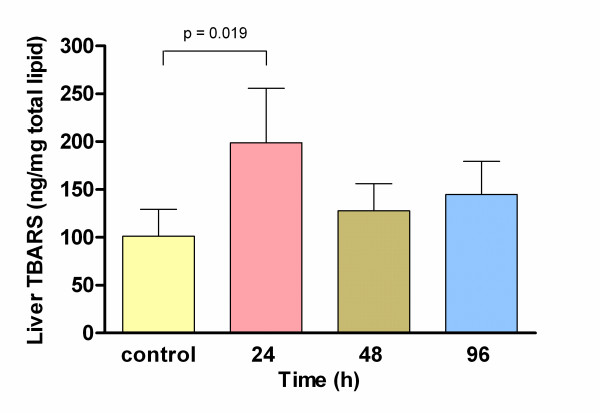
**Thiobarbituric acid reactive substances liver levels in control group and 24, 48 and 96 h after ELF-EMF exposure**. A significant increase was observed at 24 h in comparison with control group by ANOVA.

There were no significant differences, in total lipids, triacylglycerols and protein values at any time (24, 48 and 96 h, data not shown). Liver total cholesterol showed a transient increase level at 24 h (48.6 ± 8.3 mg/dL) that decreased at 96 h (32.8 ± 6.1 mg/dL; p = 0.026) (Fig. 3). Furthermore, liver TBARS concentration was the variable where the ELF-EMF exposure induced major increases 24 h after beginning the treatment (198.80 ± 56.9 vs. control group 102.18 ± 27.8 ng/mg total lipids) meanwhile no significant differences were found at 48 or 96 h (Fig. 4).

## Discussion

Extremely low frequency electromagnetic fields interact with an animal by inducing electric fields inside the body. These induced fields represent the internal exposure or "dose" [31]. In living animals, a variety of natural endogenous electric fields also exist internally. These fields arise from normal physiological activity, and extend into adjacent tissues throughout the body. The endogenous fields will combine by simple addition with any field induced by external exposure to electromagnetic fields.

Some studies about biological effects due to 50–60 Hz electromagnetic fields exposure have been performed with rodents and other animal models [32,33]. Assessment of possible human health effects has been to a certain extent supports on these studies. It has been recognized that induced electric field in rodents are much lower than in humans for the same exposure field [34], this is a direct result of differences in size [35].

Since *in situ *measurement of induced field cannot be performed in humans, dosimetric models and animal studies are used. Comparisons of all dosimetric models are agreed within 2% or less; however, differences dues of size, shape, postural and individual organ have been observed [36].

In the present study, we use the rodent model to test the time-course effects of a single exposure to ELF-EMF (60 Hz, 2.4 mT, by two hours) on serum and liver lipids, as well as liver lipoperoxide content. The whole body exposure approach could stimulate any tissue; however, among the most susceptible tissues to EMF exposure are brain, blood and liver [37]. No changes were observed in total cholesterol and triacylglycerol serum levels. But HDL-C and free fatty acids, were higher at 48 and 24 hours, respectively, in stimulated group than in sham-stimulated; however, only HDL-C showed lower levels at 96 hours after beginning the exposure. Our results are partially in accordance with previous studies showing that a single one hour exposure to a 12 Hz, 1.5–12 mT pulsed magnetic fields decreased, in a reversible way, cholesterol and triacylglycerol plasma levels in male rats 24 hours after their exposure [29,38]. Furthermore, when hyperlipidemic diet-induced female rabbits were exposed to a 15 Hz, 0.4 mT, 10 hours/day by 8 weeks, both cholesterol and triacylglycerols were significantly decreased, while HDL-C values were increased [28]. Harakawa and colleagues [37] observed that the exposition for 14 days to ELF-electric fields of ischemic rats significantly decreased free fatty acids and triacyglycerol plasma levels. The fact that total cholesterol and triacylglycerol serum levels did not change in our study could be explained in part by the frequency and length of stimulation used, as well as the basal lipemia of animals. Because of changes in serum free fatty acid concentration under our experimental conditions were observed at 24 h, it can be proposed that ELF-EMF have early effects on adipocytes lipolysis, at least after a single EMF stimulation.

On the other hand, TBARS levels were also increased in experimental group at 24 hours after the exposure. Because of the nitric oxide (NO) synthase may be stimulated by the EMF exposure [25-27], and this compound has been involved in the stimulation of lipid peroxidation during the initial states of ischemia-reperfusion injury [39], it is possible that increased free fatty acid deposition in the liver after ELF-EMF could occur as a consequence of the NO production. This hypothesis could explain also the increased TBARS production in stimulated group.

Lipid peroxidation products have been accepted as biomarkers for oxidative stress in biological systems [40]. Furthermore, few studies have been focused on the involvement of oxidative stress in the action mechanism of EMF exposure [41,42]. Some authors have showed that exposition to electric fields (50 Hz) of Sprague-Dawley rats did not change the antioxidant activity and lipid peroxide level of unstressed animals but decreased the plasma peroxide level in stressed rats [43]. The authors suggest that the electric fields might have some influence on lipid peroxide metabolism. In the present study we also obtained changes in liver peroxide level when Wistar rats were exposed to electromagnetic fields. Recently, Zwirska-Korczala and colleagues [44] using preadipocytes, showed a diminution in the activity of superoxide dismutase after 24 and 48 h of EMF exposition (180–195 Hz). The early increase in the liver TBARS concentration observed in our study after 24 h of EMF stimulation could be the result of the reduction in the activity of antioxidant enzymes or/and the increase of free radical production.

No differences were observed in cholesterol content of the liver between both groups studied. This result contrast with those finding in other studies [28,29], but could be explained by the different stimulation conditions used in that studies.

On the other hand, reduction in serum total lipids has also been observed in human beings. The most pronounced changes were found in steelworkers with the longest exposure (over 10 years) to electromagnetic fields [45].

## Conclusion

In conclusion, the results described here demonstrate the adaptative temporary response on lipid metabolism after the single exposure to ELF-EMF. The increased TBARS level after a single exposure to ELF-EMF deserves more research in other ELF-EMF conditions. Although the mechanisms for the effects of ELF-EMF are not well understand yet, the effects described above could be useful in the comprehension of lipid changes observed during chronic exposure to ELF-EMF.

## Methods

### Reagents

All reagents and chemicals used were of analytical grade (Sigma-Aldrich, México). Solvents were purchased from Merck (México). Total cholesterol (TC), high-density lipoprotein cholesterol (HDL-C), triacylglycerols (TAG), and free fatty acids (FFA) were assessed by enzymatic kits (Roche, México).

### Animals

Forty male rats of the Wistar strain, weighing 220–250 g were used. All animals were housed per group (five animals per acrylic cage) with free access to food and water, in a room with controlled temperature (25 ± 2°C) and light-dark cycles (07:00 – 19:00 h, light on). All experiments were conducted during the light phase of the cycle, between 09:00 and 15:00 h.

All procedures were performed in strict accordance with the international guidelines for care of experimental animals.

The rats were either exposed to a single stimulation of ELF-EMF (experimental groups) or sham-stimulated (control groups). The time-course effects of the single ELF-EMF exposure on serum and liver lipid concentrations, as well as liver lipoperoxides (measured as TBARS) were assessed at 24, 48 and 96 h after beginning the treatment (five rats per group at each time).

### ELF-EMF exposure

Electromagnetic field exposure was applied with a device used previously in our laboratory [46]. Electromagnetic fields were generated inside the exposure chamber with a pair of circular Helmholtz coils (30 cm internal diameter) composed of 18-gauge copper wire (350 turns). The two coils were connected in parallel to minimize the total impedance of the wire and to map the magnetic field. Coils were connected to a 120 V adjustable transformer (Staco Energy Products, Dayton, OH, USA). An oscilloscope (Tektronix, 5103N, USA) was coupled to the system for monitoring the 60 Hz sinusoidal MF waveform. Magnetic flux density was measured using a hand-held Gauss/Tesla Meter (Alpha-Lab). The sinusoidal magnetic flux density was 2.4 mT. Helmholtz coils provide a very uniform field over a relatively large volume in the space between the coils.

Coils were spaced apart at a distance equal to their radii in the upper and lower face of the plastic exposure chamber (30 × 30 × 15 cm). A single stimulation was applied during 2 h from 09:00 to 11:00 h. The exposure chamber housed five rats each experimental session. Sham-stimulated animals were maintained simultaneously to experimental animals for an equal period of time inside of another chamber with the coils turned off. The magnetic field ambient background level was <0.04 mT. Inside the exposure chamber the temperature was 25.4 ± 0.4°C and illumination intensity was 17 ± 2 Lux.

### Serum analyses

The animals were fasted twelve hours previously to the end of experimental period. The rats were anesthetized with diethyl ether and killed by cervical dislocation. The serum was obtained by blood centrifugation and stored at -78°C until triacylglycerols, total cholesterol and free fatty acids were assessed. HDL-C was determined using aliquots of fresh serum.

### Liver analyses

The liver was excised, weighed and stored at -78°C. For each liver, a sample of fresh tissue was obtained for lipid analyses.

Total lipids were extracted with chloroform-methanol (3:1 v/v) by a modified Folch's method [47]. For liver samples, 1.0 g of fresh tissue was homogenized in 4 volumes of 0.05 M phosphate buffer, pH 7.2 containing 0.025% butylated hydroxytoluene (BHT), as antioxidant. Then, the pH was adjusted to 6.0 by the addition of HCl solution and this suspension was extracted three times with 3 volumes each of the chloroform-methanol mixture. The extract was washed with 10 mL of water, the organic fraction was evaporated under a nitrogen stream, then weighed (for total lipids), and stored at -78°C until cholesterol, triacylglycerol, and TBARS analyses were performed. Liver lipoperoxides were measured by determining TBARS as previously described [48]. Total protein content was determinated in homogenate aliquots, using Bradford's method [49].

### Statistical analyses

Results were evaluated by one way analysis of variance (ANOVA) except those from serum and liver triacylglycerols (Kolmogorov-Smirnov). Differences among groups were assessed by Tukey and Mann Withney-U tests, using SPSS software v. 12. A statistical *p *value less than 0.05 was considered significant.

## Competing interests

The author(s) declare that they have no competing interests.

## Authors' contributions

PVTD participated in the collection, design, analysis and interpretation of data and writing of the manuscript; AFH participated in the collection and analysis of data and performed the statistical analysis; MAJO participated in the design, analysis and interpretation of data and writing of the manuscript; DEV participated in the design of equipment; LVD participated in the analysis and interpretation of data and writing of the manuscript. All authors read and approved the final manuscript.
